# Positive changes in the continuous desaturation index during mechanical ventilation are associated with mortality due to acute respiratory failure

**DOI:** 10.1186/cc9617

**Published:** 2011-03-11

**Authors:** G Vazquez de Anda, S Larraza, J Talavera, L De la Cruz Avila, H Lopez, D Rodriguez

**Affiliations:** 1 Universidad Autonoma del Estado de Mexico, Toluca, Mexico; 2 Hospital Materno Perinatal Monica Pretelini del Instituto de Salud del Estado de Mexio, Toluca, Mexico; 3 Centro Medico del Instituto de Seguridad Social del Estado de Mexico y Municipios, Toluca, Mexico

## Introduction

We have previously shown that the desaturation index (DI) and the continuous desaturation index (CDI) displayed on the desaturation index monitoring system (DIMS) have a high sensitivity and specificity to identify lung dysfunction [1,2]. However, dynamic changes during mechanical ventilation (MV) that may reflect the patient's response for MV treatment have not yet been tested.

## Methods

Fifty-eight patients with and without ALI/ARDS were followed during the first 24 hours of MV with the DIMS. The system computes the CDI from the positive end-expiratory pressure (PEEP), the inspired fraction of oxygen (FiO_2_) and arterial saturation by pulse oximetry (SpO_2_) [1,2]. The CDI is a percentage that is displayed graphically and numerically. Patients were divided into three groups according to the initial (first hour) CDI. Group (G) I (*n *= 16), CDI above 90%. GII (*n *= 22), CDI between 70 and 90%. GIII (*n *= 20), CDI below 70%. Then, changes in the CDI were calculated every hour (CDIh1 minus CDIh2, CDIh2 minus CDIh3, and so forth), three types of changes were expected: no change (even), negative changes (improvement of lung function) and positive changes (worsening of lung function). The mean of CDI changes was calculated at 6, 12, 18 and 24 hours after the initial recording. All patients were followed and mortality associated with acute respiratory failure (ARF) was recorded.

## Results

Changes (mean ± standard deviation) at 6 hours for GI: -1.82 ± 4.2, GII: -2.27 ± 9.2 and GIII: 2.52 ± 5.2 (*P *= 0.061). At 12 hours: GI: -2.2 ± 4.9, GII: -2.2 ± 9.1 and GIII: 6.07 ± 13.7 (*P *= 0.014). At 18 hours: GI: -1.36 ± 5.2, GII: -4.24 ± 11 and GIII: 5.47 ± 19.2 (*P *= 0.068). At 24 hours: GI: -2.09 ± 4.7, GII: -4.24 ± 12.8 and GIII: 8.53 ± 27.8 (*P *= 0.058). The mortality rate was 17.9% for GI, 33.3% for GII and 73.3% for GIII (*P *= 0.01). The association between positive changes and mortality was 30.8% for GI, and 100% for GII and GIII (*P *= 0.01).

## Conclusions

We conclude that positive changes in the CDI during the MV are associated with mortality due to ARF. The CDI may help to improve the MV settings according to the patient's response to the FiO_2 _and PEEP treatment.
